# Treatment of Rotator Cuff Tears with platelet rich plasma: a prospective study with 2 year follow‐up

**DOI:** 10.1186/s12891-021-04288-4

**Published:** 2021-05-29

**Authors:** Chadwick C Prodromos, Susan Finkle, Alexandra Prodromos, Jasmine Li Chen, Aron Schwartz, Lucas Wathen

**Affiliations:** Illinois Sportsmedicine and Orthopaedic Centers, 1714 Milwaukee Ave, 60025 Glenview, IL USA

**Keywords:** Platelet rich plasma, Rotator Cuff tear, Rotator Cuff partial tear

## Abstract

**Background:**

Surgical treatment of full-thickness rotator cuff (RC) tears is associated with generally good results. There is no consensus regarding treatment of partial thickness tears that fail conservative treatment. The purpose of this study was to look at the efficacy and confirm the safety of dual injection PRP into the shoulder of patients with rotator cuff pathology who have failed conservative treatment with followup to two years.

**Methods:**

Seventy-one shoulders with MRI confirmed, rotator cuff pathology who failed conservative treatment, had dual PRP injection into the rotator cuff. Global improvement, Quick DASH and VAS scores were collected at 6, 12, and 24 months after treatment and comparison of means was used to analyze changes.

**Results:**

No adverse events were seen in any patient. Based on global rating scores positive results were seen in 77.9 % of patients at 6 months, 71.6 % at 1 year, and 68.8 % of patients at 2 years. Mean VAS scores improved from 50.2 [CI 44.4–56.0] pre-injection to 26.2 [CI 19.5–32.9] at 6 months, 22.4[CI 16.1–28.7] at 1 year and 18.2 [CI 12.3–24.1] at 2 years (p < 0.0001 for all). The mean Q- DASH scores (0-100, 100 worse) improved from 39.2 [CI 34.3–44.1] for all patients before treatment to 20.7[CI 15.0-26.4] at 6 months, 18.0[CI 12.9–23.1] at 1 year, and 13.8 [CI 8.4–18.8] at 2 years (p < 0.0001 for all). No patient with partial tear had clinical evidence of progression to full thickness tear. When separated into subgroups based on rotator cuff status, all subgroups showed improvement. Patients in the > 50 % partial tear group had the best overall improvement based on Global Rating scores while those in the tendinitis group had the poorest outcomes.

**Conclusions:**

PRP injection is a safe and effective treatment for RC cuff injury in patients who have failed conservative treatment of activity modification and physical therapy without deterioration of results two years after treatment. Better results are obtained with greater structural tendon damage than in shoulders with inflammation without structural damage.

**Trial registration:**

This is not a clinical trial.

**Supplementary Information:**

The online version contains supplementary material available at 10.1186/s12891-021-04288-4.

## Introduction

Surgical treatment of full thickness rotator cuff (RC) tears is associated with generally good results [1]. There is no consensus regarding treatment of partial thickness tears that fail activity modification and physical therapy [2]. Three types of surgical treatment can be performed, debridement with or without acromioplasty, attempted repair of a flap of partial thickness tendon tear to the footprint, or conversion of the partial tear to a full thickness tear and subsequent repair. Debridement alone has a high reoperation rate, especially in smaller partial tears [3, 4]. Attempted repair of a flap of partial thickness tendon tissue back to the footprint is technically difficult due to the small space allowed under the remaining tendon and can damage the remaining tendon. Results of this technique have not been consistently good. [5] Conversion to a full tear requires intentionally cutting remaining intact tissue and can lead to stiffness and a high re-tear rate [6, 7]. Corticosteroid injections are sometimes used, but have been associated with tendon damage [8–10]. Platelet rich plasma (PRP) however has been shown to enhance connective tissue healing in patellar tendons [11]. In the shoulder, PRP has been used for treatment of RC tendinitis or partial thickness RC (PTRC) tears in a number of studies [12–23] and has shown improvement in symptoms compared to steroids [18, 19, 21, 22], physical therapy [13], hyaluronic acid [12], prolotherapy [17, 18], and placebo controls [12, 15]. Most of these studies have a short follow up time of 6 months or less, with only a few extending follow up to a year [12, 13, 15, 20]. Some years ago we began injecting the shoulder with PRP from an anterolateral approach into the critical zone of the rotator cuff and sub-acromial bursa. While most patients improved, many did not. We felt that due to the curvilinear nature of the tendinous insertion we were probably missing areas of pathology more proximally, especially on the articular side of the tendon. We therefore began to add a second injection from a posterior glenohumeral approach, essentially the posterior arthroscopic portal area. Injection in this area bathes the supra and infraspinatus tendons with PRP from the equator of the humeral head proximally toward the glenoid, an area that we felt our anterolateral injection was missing. In addition, in the presence of a patent rotator cuff, the shoulder has two distinct, non-communicating compartments (bursal and articular) and dual injection allows treatment of both compartments. We noticed an immediate improvement in clinical results when we adopted this dual injection technique and adopted this as our standard treatment.

The purpose of this study is to look at the efficacy and confirm the safety of this dual injection PRP protocol in the treatment of patients with rotator cuff pathology who have failed conservative treatment. Secondary aims are to see if patients with more severe rotator cuff damage would respond better or worse to treatment than patients with less severe damage and to assess whether treated patients would develop clinical symptoms indicating complete rupture of the rotator cuff.

## Methods

Beginning in January of 2015, dual PRP injection was offered to all patients who had failed activity modification and physical therapy for rotator cuff pathology. All patients seen in this practice and diagnosed with rotator cuff pathology are initially placed on conservative treatment including activity modification and physical therapy. A patient is considered to have failed conservative treatment if they have not shown any improvement in symptoms after 1 month of treatment, or if after several months and despite some initial improvement, improvement with treatment has stopped but the patient still has significant symptoms of pain and disability. Standard activity modification in our practice includes:

1. Avoidance of non-steroidal anti-inflammatory medication (NSAIDs) since there is evidence that NSAIDs, both Cox 1 and 2, interfere with rotator cuff healing [24, 25].
2. Avoidance of corticosteroid injection into the shoulder, because corticosteroids can cause tendon damage [8–10].
3. Avoidance of all activities that cause pain, since pain is an indication of further tendon damage.
4. Avoidance of other analgesics including topical liniments, ice, kinesiotape, and oral analgesics, which can accelerate damage by masking pain and allowing greater use. We counsel patients that the “pain is their friend” because it tells them what not to do and should not be masked. An exception is made for acetaminophen which can be used sparingly if necessary.
5. Maintenance of the shoulder at less than 45 degrees of elevation during activities to minimize further stress to the rotator cuff.

Rotator cuff pathology was established by clinical examination and confirmed with MRI in each case. The lead author performed all clinical examinations and rotator cuff pathology was diagnosed based on anterolateral shoulder pain, a positive NEER and Hawkins sign, and increased pain with elevation of the arm. All patients included in the study had active symptoms including pain, especially with use of the shoulder. MRI was performed by third-party facilities and the results were read by radiologists at those facilities. High signal in the supraspinatus was present in most patients, but was not necessary for clinical diagnosis of rotator cuff pathology. MRI reports included information about the extent of rotator cuff tearing if present. Based on the MRI results, patients were separated into 4 subgroups: the Tendinitis group (patients with rotator cuff pathology on exam but no apparent rotator cuff tear on MRI), the Partial Tear < 50 % group (patients with rotator cuff pathology on exam and shoulders with less than 50 % thickness supraspinatus tendon tears on MRI), the Partial Tear > 50 % group (patients with rotator cuff pathology on exam and greater than 50 % thickness supraspinatus tendon tears on MRI), and the Full Thickness Tear group (patients with rotator cuff pathology on exam and full thickness supraspinatus tendon tears on MRI). We generally recommend surgical repair for full thickness tears, so most full-thickness tear patients opted for surgery and were therefore excluded from this study. However, there were no other inclusion or exclusion of patients with full thickness tears based on the size of the tear. Any patient who had a full thickness rotator cuff tear who was not a surgical candidate and who failed conservative treatment was offered inclusion in this study. Patients who agreed to injection had proper informed consent obtained and were prospectively enrolled into the study. Patients who had surgery or other treatment in the six months prior to the PRP injection or who had other significant shoulder pathology, such as severe arthritis, were excluded from the study.

All patients had AP, Grashey (true AP) and Y views x-rays of the shoulder as well MRI scan before injection. Quick Disabilities of the Arm, Shoulder, and Hand (Q-DASH) assessment [26] and Pain Visual Analog Scale (VAS) [27] scores were obtained on all patients immediately prior to treatment.

**Fig. 1:**
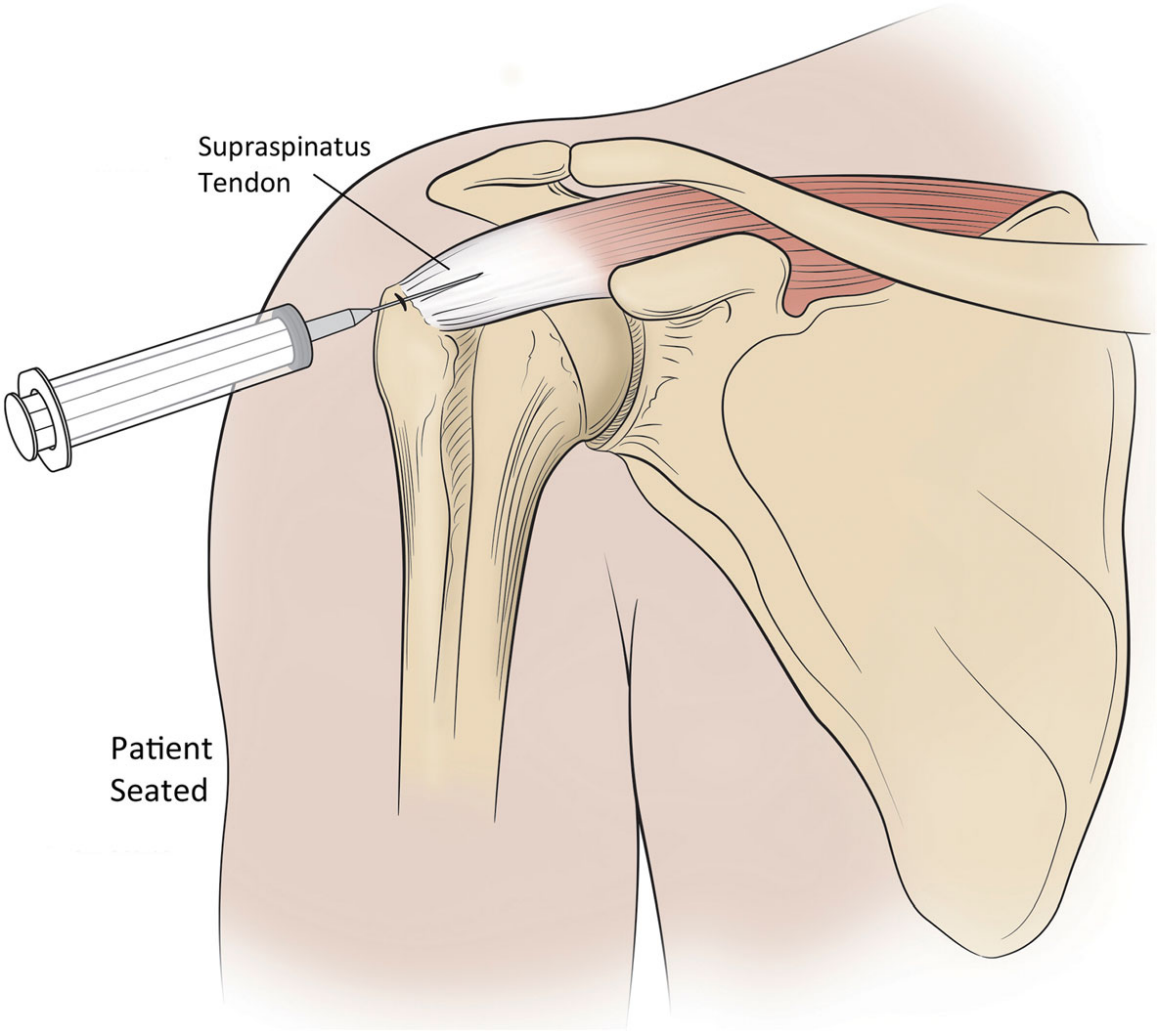
First injection was made into the supraspinatus tendon insertion critical zone and bursal area with the patient seated

**Fig. 2:**
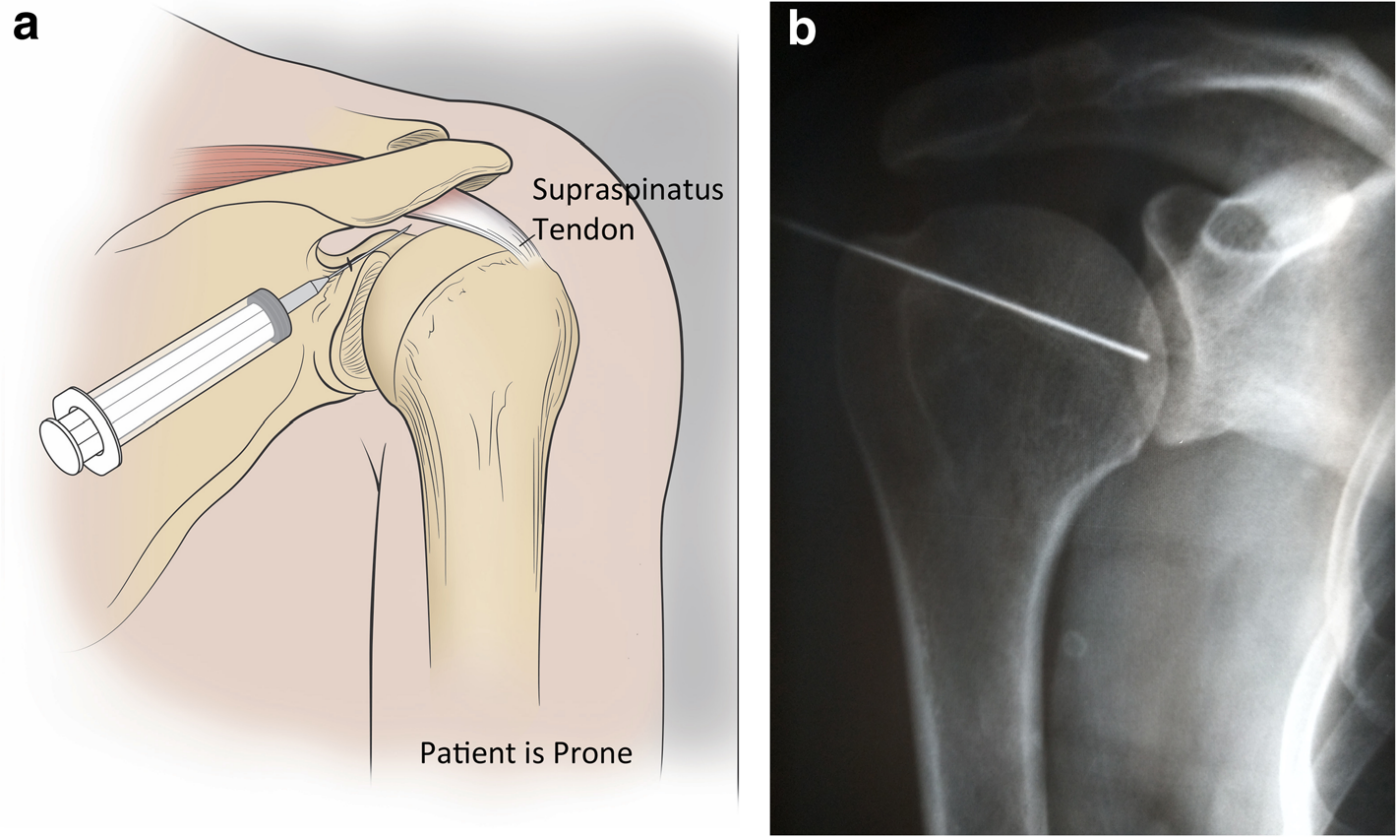
**a** Second injection was made into the glenohumeral intra-articular space under the supraspinatus tendon at or just proximal to the superior equator of the head while patient was prone. **b** X-ray image shows needle placement for the second injection

90ml of blood was drawn from each patient and processed through a double spin technique to create two 4ml doses of PRP. According the PAW (Platelets/Activation/White cells) classification system, the PRP preparation was P3-Aα [28]. PRP was injected in 2 separate locations at the time of treatment under ultrasound guidance. The first injection was into the supraspinatus tendon insertion critical zone and bursal area with the patient seated. (Fig. 1) No lidocaine or other anesthetic was used to avoid tendon damage and the known inhibition of PRP effect from -caine anesthetics [29, 30]. The second injection was performed with the patient prone into the glenohumeral intra-articular space under the supraspinatus tendon at, or just proximal to, the superior equator of the humeral head. 3 cc of 1 % lidocaine was injected down to but not through the capsule of the shoulder prior to this PRP injection. (Figure 2a and b)

After treatment, patients were advised to limit activity for one week, and to use topical ice and acetaminophen as needed. After one week, patients were counseled to resume normal activities but to continue with activity modifications as instructed before treatment.

Additional injections were performed subsequently in a few patients at follow-up to enhance the result of the first injections, as a result of shared clinical decision making with the patient. Clinical outcome and pain were evaluated using patient completed Q-DASH assessment and VAS at 6 months, one year and 2 years after treatment. A global assessment of combined pain and functional improvement was performed by asking patients for a percent improvement from before treatment to the follow up point. Patients who described at least a 30 % improvement were considered to have a significantly improved outcome.

Statistical analysis was performed using comparison of means between the pre-treatment VAS and Q-DASH scores and scores at 6 months, 1 year and 2 year. Similar analysis was performed for scores in each of the subgroups and for the change in global scores between subgroups. The means and the standard deviations for each subgroup were then used to calculate with a 95 % confidence the confidence interval of the results calculated. A comparison of means was performed between the four subgroups based on the mean ages and gender of each group. The percent of patient significantly improved in each subgroup was analyzed using Chi square comparisons between the 4 subgroups.

## Results

Eighty-two patients (85 shoulders) with confirmed rotator cuff pathology were treated with PRP dual injections. Fourteen patients were excluded: 4 who had surgery within six months prior to PRP injection, 1 who had stem cell injection within 6 months prior to injection, 2 with severe glenohumeral arthritis in addition to the rotator cuff pathology, 1 with intra-articular loose bodies, and 6 with significant SLAP Type II lesions. This left 68 patients (71 shoulders) in the study. The subgroups based on MRI results included 20 shoulders with tendinitis, 27 shoulders with partial thickness tear of < 50 %, 14 shoulders with partial thickness tears of > 50 %, and 6 shoulders with full thickness tears. See Table 1. The full thickness tears ranged from 1 pinhole tear, 3 small to moderate tears with no retraction, 1 large tear with slight retraction and 1 large tear involving both the supraspinatus and infraspinatus with retraction and some atrophy. The age range was from 23 to 86 years with a mean of 51.7 years (standard deviation = 16.2). There were 37 males (39 shoulders) and 31 females (32 shoulders). Comparison of means shows that there are statistically significant differences between the groups based on age with tendinitis group being significantly younger and the full-thickness tear group significantly older than the mean age (p = 0.01 and p = 0.02 respectively). These differences are not unexpected. Younger patients are much more likely to have tendinitis than actual rotator cuff tearing. Full thickness rotator cuff tears are most common in older patients. No significant differences in gender were seen between the subgroups.

**Table 1:** Patient Demographics

Demographics	All Shoulders – Joints (Patients)	Tendinitis	Partial Tear < 50 %	Partial Tear >50 %	Full Thickness Tear
Total	71 (68))	20	27	18	6
Male	39 (37)	14	16	6	3
Female	32 (31)	6	11	12	3
Mean Age	51.7	41.1	55.9	51.6	68.3

Thirteen patients received a second set of dual injections ranging in time from 10 days to 20 months after the initial injection (median time 4 months). Two patients received 2 additional dual injections; one at 1 and 5 months, the other at 6 and 20 months after the index injections. All other patients received only the initial dual injections. Four patients sought alternate treatment from other physicians after their PRP injections. Two received a shoulder corticosteroid injection at 5 months and 7 months post PRP injection, one had a Tenex procedure (a non-surgical procedure for the removal of scar tissue) performed for tendinitis 10 months after injection. One patient had a total shoulder replacement 18 months after treatment,despite having only tendinitis and minimal osteoarthritis. Although no follow up MRIs were performed, no patient was found to develop worsening symptoms indicative of a full thickness rotator cuff tear after treatment that did not have one before treatment.

Of the three patients who had bilateral injections, two of them had bilateral tendinitis and one had tendinitis in one shoulder and a > 50 % partial thickness tear in the other. Outcomes for each shoulder were reported separately. One of the patients with bilateral tendinitis had good results out to 2 years. The other had a good results in one shoulder at 6 months but was back to baseline in both shoulders by 1 year. The third patient had a better outcome in the shoulder with a > 50 % tear with results good to 2 years. The tendinitis shoulder was back to baseline by 2 years.

There were no infections or other adverse events of any kind at any time in any shoulder after injection. Most patients had moderate, self limited soreness generally lasting for about one week and were counseled to expect this. Only acetaminophen and ice were used and no patients required other analgesics.

Sixty-eight shoulders (95 %) were available for evaluation at 6 months after injection, 67 shoulders (94 %) at 1 year, and 64 shoulders (90 %) at 2 years. The magnitude of Q-DASH, VAS and global improvement scores are shown in Table 2.

**Table 2:** Global Improvement, VAS and Q-DASH Total Scores and Groups

			Pre Treatment	6 Months	1 Year	2 Years
**Global Improvement (Percent Improvement)**	All Patients	#	-	68	67	64
		Mean	-	58.5	60.6	58.5
		StDev	-	36.9	40.5	41.1
	Tendinitis	#	-	19	18	15
		Mean	-	43.4	39.4	31.3
		StDev	-	41.6	43.3	42.1
	< 50 % Partial Tear	#	-	25	25	25
		Mean	-	65.1	68.3	62.7
		StDev	-	36.3	39.5	43
	> 50 Partial Tear	#	-	18	18	18
		Mean	-	64.1	70.8	74.1
		StDev	-	31.6	36.3	30.4
	Full Tear	#	-	6	6	6
		Mean	-	61.7	60.8	61.7
		StDev	-	33.1	32.6	33.1
**VAS**	All Patients	#	64	62	61	53
		Mean	50.2	26.2	22.4	18.2
		StDev	23.1	26.4	24.6	21.5
		Change in Mean	-	24	27.8	32
	Tendinitis	#	18	16	16	10
		Mean	46.9	27.8	35.3	24
		StDev	26.2	30.6	30.6	31
		Change in Mean	-	19.1	11.6	22.9
	< 50 % Partial Tear	#	25	23	22	22
		Mean	51.1	27	13.4	13.3
		StDev	21.4	28.2	16.6	17.6
		Change in Mean	-	24.1	37.7	37.8
	> 50 Partial Tear	#	16	17	17	16
		Mean	46.9	23.3	21.5	20.3
		StDev	23.4	23	24.4	20.6
		Change in Mean	-	23.6	25.4	26.6
	Full Tear	#	5	6	6	5
		Mean	68	25	23.3	22
		StDev	13	20.7	21.6	19.2
		Change in Mean	-	43	44.7	46
**Q-DASH**	All Patients	#	68	60	59	53
		Mean	39.2	20.7	18	13.8
		StDev	20.8	22.4	19.8	18.8
		Change in Mean	-	18.5	21.2	25.4
	Tendinitis	#	19	15	15	10
		Mean	38.8	24.2	30.2	14.3
		StDev	22.5	27.4	23.4	19.7
		Change in Mean	-	14.6	8.6	24.5
	< 50 % Partial Tear	#	27	23	22	22
		Mean	38.7	18.6	10.5	9.8
		StDev	21.4	21.2	12.7	15.2
		Change in Mean	-	20.1	28.2	28.9
	> 50 Partial Tear	#	17	17	17	16
		Mean	34.6	17.9	14.1	18.7
		StDev	16.5	19.6	17.6	24.7
		Change in Mean	-	16.7	20.5	15.9
	Full Tear	#	5	5	5	5
		Mean	59	29.6	28.2	14.2
		StDev	18.5	24	22.7	5.4
		Change in Mean	-	29.4	30.8	44.8

**Fig. 3:**
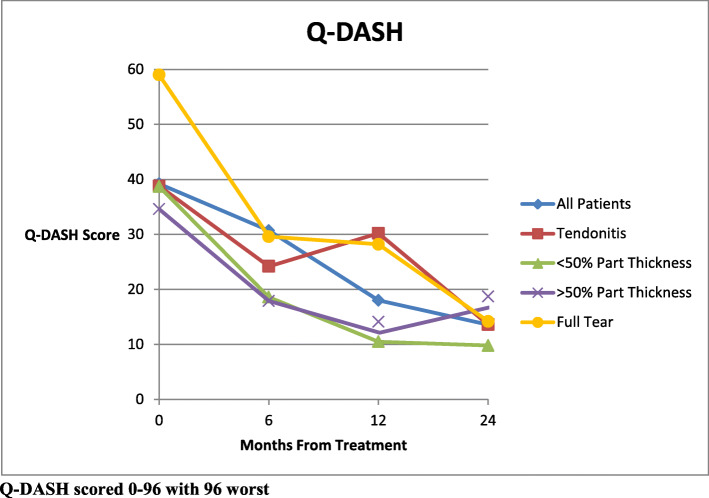
Q-DASH Scores from Pre-Treatment to Follow Up: Greater decline indicates greater clinical improvement

**Fig. 4:**
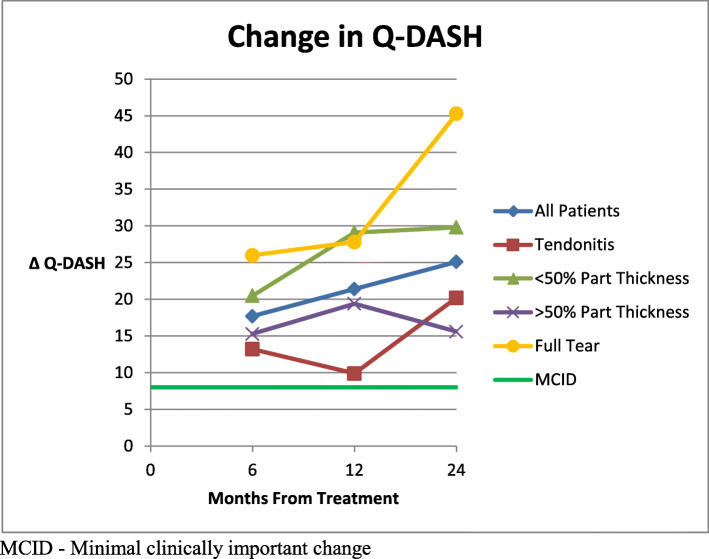
Mean change in Q-DASH scores from pre-treatment compared to MCID: greater increase indicates greater clinical improvement

The mean Q- DASH scores (0-100, 100 worst) improved from 39.2 [95 % CI 34.3–44.1] for all patients before injection to 20.7 [95 % CI 15.0-26.4] at 6 months, 18.0 [95 % CI 12.9–23.1] at 1 year and 13.6 [95 % CI 8.84–18.8] at 2 years (p < 0.0001 for all follow up times) post treatment, showing a steady improvement over time. (Fig. 3) The improvement in the Q-DASH for both partial tear groups was statistically significant at all follow up times. The full tear group Q-DASH scores were statistically improved at 1 year and 2 years, while the 6 month result showed improvement but was not statistically significant (p = 0.06). In the tendinitis group, only the 2 year follow up scores were significantly improved from the pre-treatment scores (p = 0.01). Comparisons of Q-DASH scores were made between groups at all follow up intervals. The < 50 % and > 50 % partial tear group scores at the 1 year follow up interval were significantly better than the tendinitis scores (p = 0.003 and p = 0.004 respectively). No other significant differences were found between any other groups at the 6 month, 1 year, or 2 year follow up intervals. A change in mean Q-DASH results from before injection to each follow up point was calculated. Mintken [26] reported that the minimal clinically important change (MCID) for Q-DASH scores is 8. All mean follow up Q-DASH scores were above this MCID. The change in scores is illustrated in Fig. 4 along with a line indicating the MCID. Comparison of means between groups showed a significant difference between the tendinitis group and the < 50 % partial tear group at 1 year (p = 0.01). All other differences were not statistically significant.

**Fig. 5:**
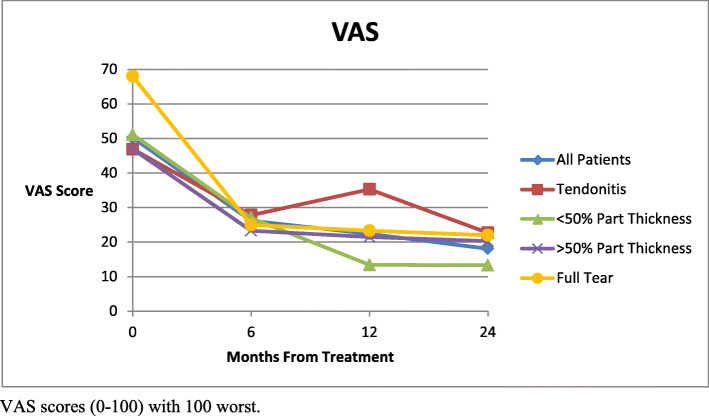
Change in VAS Scores from pre-treatment to follow up: lower score indicates greater clinical improvement

**Fig. 6:**
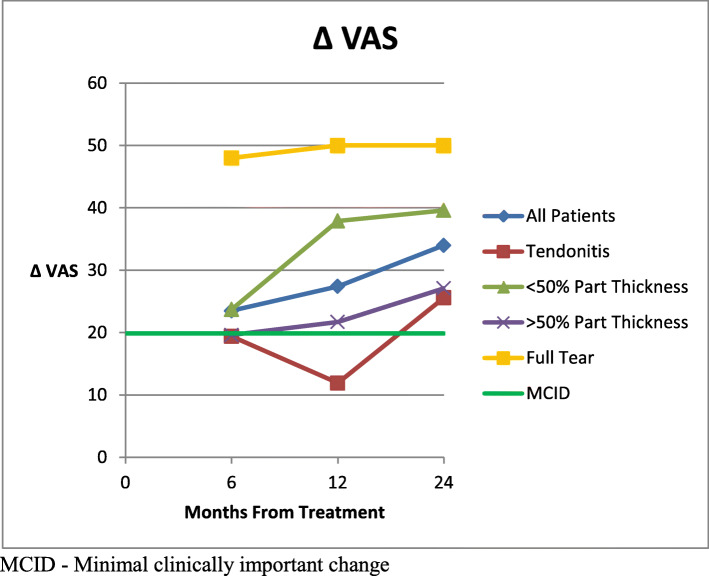
Mean change in VAS scores from pre-treatment compared to MCID: greater score indicates greater clinical improvement

Mean VAS scores (0-100, 100 worst) significantly improved from 50.2 [95 % CI 44.4–56.0] before injection to 26.2 [95 % CI 19.5–32.9] at 6 months, 22.4 [95 % CI 16.1–28.7] at 1 year, and 18.2 [95 % CI 12.3–24.1] at 2 years (p < 0.0001 for all follow up times) post injection. (Fig. 5) Within the subgroups, the VAS scores were significantly improved from Pre-treamtent scores to all follow up times in the < 50 % partial tear group, the > 50 % partial tear group and the full tear groups. Within the tendinitis group, there were improvement trends in the mean VAS score at each follow-up time but none were statistically significant compared to pre-treatment levels. Comparisons of VAS scores were made between groups at all follow up intervals. The only significant difference found was a better outcome in the < 50 % partial tear group than the tendinitis group at the 1 year follow up time (p = 0.007). A change in mean VAS results from before injection to each follow up point was calculated. Tubach [31] reported that MCID for VAS scores is 19.9. The mean follow up scores for the tendinitis group for 6 months and 1 year were below this MCID, although the two year result was above the MCID. All results at all time points for the partial tear groups and the full thickness tear group were above the MCID. The change in scores is illustrated in Fig. 6 along with a line indicating the MCID. Comparison of means of the change in VAS scores between groups showed a significant difference between the tendinitis group and the < 50 % partial tear group at 1 year (p = 0.04). All other differences were not statistically significant.

The mean change in Q-DASH scores and the mean change in VAS scores were also broken down based on age (< 35 years old, 35–49 years old, 50–59 years old, 60–69 years old, 70 years old and up) and analyzed using comparison of means. There were no statistically significant differences between any of the groups at any time point.

**Fig. 7:**
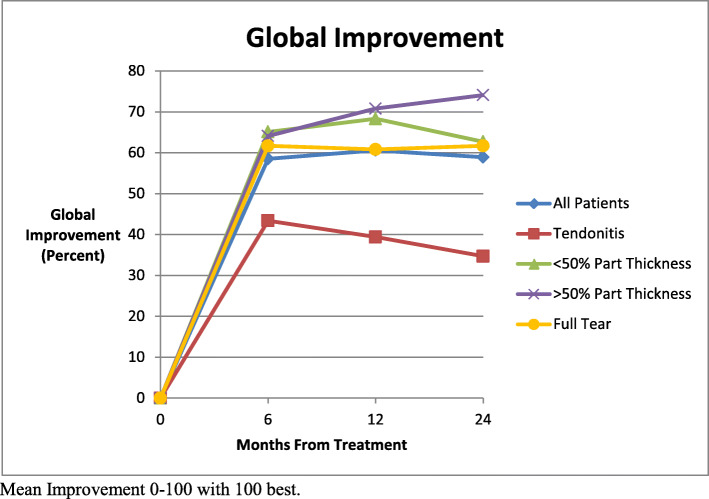
Mean global improvement from pre-treatment to follow up

Mean global improvement was 58.5 % improvement at six months, 60.6 % improvement at 1 year and 58.5 % improvement at 2 years post injection. (Fig. 7) The mean improvement of the tendinitis group was lower than all other groups at all time periods. Comparison of means between groups showed that both the < 50 % partial tear (p = 0.03 both years) and the > 50 % partial tear groups at one year and two years were significantly better than the tendinitis group scores (p = 0.02 at 1 year, p = 0.002 at 2 yrs).

**Fig. 8:**
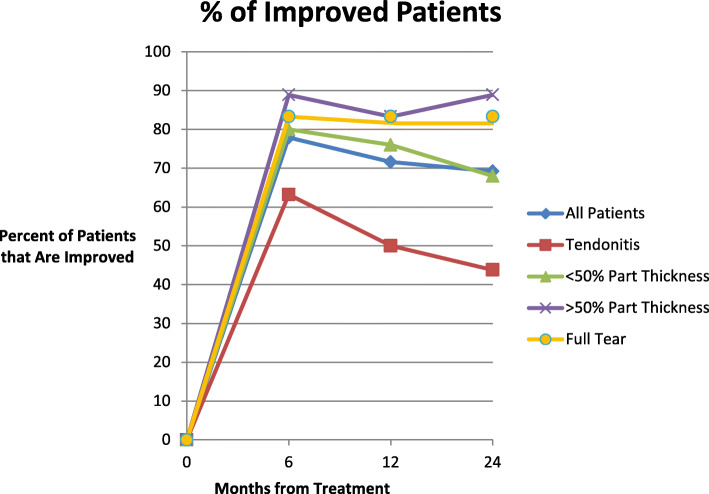
Percent of improved patients

Patients were categorized as being significantly improved if they indicated a 30 % or greater overall global improvement: combining pain and function. (Table 3; Fig. 8). Overall, 77.9 % patients were improved at 6 months, 71.6 % were improved at 1 year, and 68.8 % were improved at 2 years. The > 50 % partial tear group in general had the best outcomes with 88.9 %, 83.3 and 88.9 % of patients showing significant improvement at 6 months, 1 year and 2 years respectively. The outcomes for the full thickness tear group and < 50 % partial tear group were close to the > 50 % partial tear group. The tendinitis group had the least improvement at 63.2 %, 50.0 and 40.0 % respectively. Chi square comparisons between groups showed that the > 50 % partial tear group at one year and 2 years had significantly more improved patients than the tendinitis group (p = 0.03 and p = 0.02 respectively). All the other group comparisons were not statistically different.

**Table 3:** Improved and Unimproved Patients by Subgroup

		6 Months	1 Year	2 Years
**Overall Outcome**	All Patients	68	67	64
	Improved	53	48	44
	Not Improved	15	19	20
	% Improved	77.9 %	71.6 %	68.8 %
**Tendinitis**	All Patients	19	18	15
	Improved	12	9	6
	Not Improved	7	9	9
	% Improved	63.2 %	50.0 %	40.0 %
**< 50 % Partial Tear**	All Patients	25	25	25
	Improved	20	19	17
	Not Improved	5	6	8
	% Improved	80.0 %	76.0 %	68.0 %
**> 50 Partial Tear**	All Patients	18	18	18
	Improved	16	15	16
	Not Improved	2	3	2
	% Improved	88.9 %	83.3 %	88.9 %
**Full Tear**	All Patients	6	6	6
	Improved	5	5	5
	Not Improved	1	1	1
	% Improved	83.3 %	83.3 %	83.3 %

## Discussion

The most important findings of this study are that dual rotator cuff PRP injection is safe and produces consistently beneficial results in patients with partial rotator cuff tears that have failed conservative treatment The results continued to show benefits at two years after the initial injection. None of the treated patients presented with an increase in symptoms that would indicate progression of rotator cuff pathology from partial to full thickness tear. This is the first study to show sustained improvement out to two years post injection. It is the first to report dual injection of the rotator cuff. In our opinion, this study helps establish PRP injection as the preferred treatment for partial rotator cuff tears that fail activity modification and physical therapy. Adverse events are essentially unknown and we saw none beyond short-term pain immediately after injection. The only impediment to care is that the patients must pay for the procedure since it is not reimbursed by commercial insurance, although some worker’s compensation boards will authorize payment.

Although this study did not use a control group, all patients failed initial conservative treatment including activity modification and physical therapy before being enrolled in this study. Although conservative treatment continued after treatment with PRP, because of this earlier failure to improve, the patients worked as their own control group. We feel that we can say with a good degree of confidence that the improvement seen after the PRP injection was related to the injection and not to the conservative treatment.

A weakness of the study is the use of global improvement scores to help determine improvement of patients after treatment. This score is self-reported by patients and due to the extended follow-up time, may be subject to some recall bias by patients. We have backed up these scores with the standardized VAS and Q-DASH scores, which demonstrate results that parallel the global improvement scores. Clinically, we have found the global improvement score to be a very simple yet useful measure to track patient improvement or lack of improvement to treatment.

An additional weakness of this study is that some of the patients received 1 or 2 additional PRP injections after the initial pair. PRP was only repeated in patients who were already showing improvement from the treatment but who desired a stronger improvement. Patients who had no improvement from the initial treatment were not injected a second time. So though repeat injection may have affected the duration or degree of improvement, it did not affect whether or not a patient had a positive response to treatment. Because of this, we felt that these patients should remain included in the study. The other major finding of our study was that the injections performed best in the more structurally damaged tendons. Severe partial tears, which we worried might not benefit, did quite well overall and had the best results of any group. Less severe partial tears also did well, as did full thickness tears. Treatment of these groups, all of which had significant structural damage to the tendon, produced results that were not significantly different from each other.

However, although patients who had inflammation with minimal or no structural damage had overall benefits, they were statistically significantly less than the patients with torn tendons. This was an unexpected result, as we expected the best results in the least damaged tendons. We cannot fully explain this finding. It may be that these tendinitis patients had pain from some source other than the rotator cuff: although none had frozen shoulder, significant arthrosis, AC joint inflammation or significant labral pathology. We think it is more likely that such patients simply have enhanced sensitivity to shoulder use. This would render them symptomatic at an earlier stage of tendon wear, and also less likely to benefit from treatment. This also parallels the finding that patients with more normal pre-operative radiographs do less well after joint replacement than patients with worse pathology. [32–34]

It is interesting also that the percentage of significantly improved patients were steady or improved in the > 50 % tear and full thickness tear group at 2 year follow-up, decreased a little in the < 50 % tear group and decreased more in the tendinitis group. This strengthens the relationship between structural damage and beneficial PRP effect. The < 50 % group may be a mix of significant structural damage and high signal inflammation whose results were in fact intermediate between the definitely significantly damaged > 50 % partial tear and full thickness tear groups excellent results on the one hand, and the non-structurally damaged tendinitis group’s less good results on the other. Interestingly, within the tendinitis cohort there was a significant decrease in the number of patients as a percentage of the total cohort who showed improvement from the one to two year follow-up times. However, the mean improvement went up substantially. Thus, the cohort bifurcated between a subgroup with excellent improvement from the first to the second year and a group which did not improve.

The tendinitis subgroup was also significantly younger in age, suggesting that the lower scores in the tendinitis group could be partially due to decreased age. However, since patients with tendinitis made up the majority (73 %) of patients in the youngest age group of < 35 years old, it is impossible to differentiate the effects of age from pathology.

It is also fortunate that the PRP performed best in the definitely structurally affected tendons since most of these patients (at least in the United States) would have progressed on to surgery if the PRP had not been successful. Surgery was thus successfully avoided in the patients who were most at risk to have surgery recommended, had the PRP treatment not been offered. This is also gratifying since surgery for partial tears is generally unreliable with a relatively high failure rate [3, 4, 6, 7]. The PRP patients were thus not only spared the risks of surgery, but spared the significant likelihood of an unsatisfactory surgical result.

The third finding of this study is that based on symptoms, patients did not appear to have progression of partial thickness to full thickness rotator cuff tears. This finding would have been strengthened by the performance of post-treatment MRI, which was not done. However, worsening symptoms is a strong indicator of worsening pathology. Although not all patients improved with treatment, none showed a strong worsening of symptoms that would be an indication of transition from partial to full-thickness rotator cuff tear. A followup study that included post-treatment MRI is recommended to verify this initial impression.

Our two year follow-up is the longest yet reported for PRP for rotator cuff pathology. Our results showing improvement are consistent with other studies, [12–14, 16–18, 20–23]. The next longest follow-up was reported by Mautner [16] who found 81 % of patients were improved significantly at an average of 15 months. Importantly, we found that results overall continued to improve from the one to the two year mark for both Q-DASH and VAS and were unchanged for the subjective global improvement rating. This finding makes it likely that the good two year results found are probably unlikely to deteriorate at even longer follow up.

Rotator cuff disease in patients without full-thickness tears is mainly a disease based on symptoms. In a study by Miniaci [35] MRI was performed on 30 shoulders of young volunteers (ages 17 to 49) who had no shoulder symptoms and no known abnormalities. None of the shoulders were rated as normal with all rated at least some signal and 23 % rated has having high grade changes. Based on this study, clinical symptoms, not MRI changes are the determining factor in diagnosing rotator cuff disease. From this point of view, PRP is not just palliative but in some measure potentially “curative” for rotator cuff disease. The fact that tendinous tissue is capable of healing and regenerating makes this possibility all the more possible. We wish to emphasize however, that we did not obtain routine post treatment MRI scans to evaluate this possibility since there was no clinical reason to obtain them except in isolated cases.

We wish to express that we believe very strongly that the management of the rotator cuff injured shoulder beyond the PRP treatment is critically important. Specifically, the activity modifications detailed in the methods sections are essential to maintaining both general rotator cuff health and specific improvements produced by injection of PRP. While some physicians may think these restrictions to be impractical, we have found them both extremely effective and acceptable to patients when time is taken to explain to them why these modifications are important.

Regarding the full thickness tears in this study, we do not know how much these tears have progressed over the duration of the study since we did not obtain follow-up MRI scans. Health permitting, we recommend to our patients that all full thickness tears undergo surgical repair in patients under 80 years of age. However, in unhealthy patients and patients over 80 years old, we do not perform repair, as we think the risks of even minor arthroscopic surgery generally exceed the benefits in this population. Our results from the small number of full thickness tears injected with overall very good results and enduring benefit to two years has caused us to recommended PRP as primary treatment for pain to older or more infirm patients who have failed physical therapy. We do not use corticosteroid injections in this population (or for any shoulder patient except some patients with frozen shoulder) due to the risk of adverse events, worsening of tendon damage, and the generally short duration of improvement [10, 18, 36].

A limitation of this study is the limited size of the full thickness rotator cuff tear cohort. However the uniformity of benefit in this cohort somewhat mitigates this limitation. The limited size of each subgroup also limits the reliability of sub-group comparisons. A strength of the study is the high follow up rate and follow up out to 2 years after treatment.

## Conclusions

Dual PRP injection is a consistently safe and effective treatment for partial tears of the rotator cuff in patients that have failed conservative treatment consisting of activity modification and physical therapy. Use of dual PRP injections helps avoids surgery, produces benefit two years or longer after treatment, and prevents worsening symptoms indicative of complete tearing of the rotator cuff. PRP also provides good palliation of full thickness rotator cuff tears for patients who are not candidates for surgical repair for at least two years. PRP is effective for many patients with tendinitis without structural damage, but less often than for patients with MRI evidence of tendon tearing. We believe PRP injection should be considered the treatment of choice for patients with partial rotator cuff tear or inflammation who have failed physical therapy and activity modification.

## Supplementary Information

**Additional file 1.**

## Data Availability

All data generated or analysed during this study are included in this published article and its supplementary information files.
